# Multiomics-based analysis of the mechanism of ammonia reduction in *Sphingomonas*

**DOI:** 10.3389/fmicb.2024.1437056

**Published:** 2025-05-01

**Authors:** Wang Mingcheng, Liu Daoqi, Xia Huili, Wang Gailing, Liu Chaoying, Guo Yanan, Guo Aizhen

**Affiliations:** 1 National Laboratory of Agricultural Microbiology, Wuhan, Hubei, China; 2 College of Veterinary Medicine, Wuhan, Hubei, China; 3 Hubei Hongshan Laboratory, Huazhong Agricultural University, Wuhan, Hubei, China; 4 College of Biological and Food Engineering, Huanghuai University, Zhumadian, Henan, China; 5 Institute of Animal Science, Ningxia Academy of Agriculture and Forestry Sciences, Yinchuan, Ningxia, China

**Keywords:** ammonia reduction, ncd2 gene, transcriptomics, metabolomics, *Sphingomonas* ammonia reduction by Sphingomonas

## Abstract

Ammonia is the primary component of malodorous substances in chicken farms. Currently, the microbial ammonia reduction is considered a potential method due to its low cost, high safety, and environmental friendliness. *Sphingomonas* sp. Z392 can significantly reduce the ammonia level in broiler coops. However, the mechanisms of ammonia nitrogen reduction by *Sphingomonas* sp. Z392 remain unclear. To explore the mechanisms of ammonia reduction by *Sphingomonas* sp. Z392, the transcriptome and metabolome analysis of *Sphingomonas* sp. Z392 under high ammonium sulfate level were conducted. It was found that the transcription levels of genes related to purine metabolism (*RS01720*, *RS07605*, *purM*, *purC*, *purO*) and arginine metabolism (*glsA*, *argB*, *argD*, *aguA*, *aguB*) were decreased under high ammonium sulfate environment, and the levels of intermediate products such as ornithine, arginine, IMP, and GMP also were also decreased. In addition, the *ncd2* gene in nitrogen metabolism was upregulated, and intracellular nitrite content increased by 2.27 times than that without ammonium sulfate. These results suggested that under high ammonium sulfate level, the flux of purine and arginine metabolism pathways in *Sphingomonas* sp. Z392 might decrease, while the flux of nitrogen metabolism pathway might increase, resulting in increased nitrite content and NH_3_ release. To further verify the effect of the *ncd2* gene on ammonia removal, *ncd2* was successfully overexpressed and knocked out in *Sphingomonas* sp. Z392. *ncd2* Overexpression exhibited the most ammonia reduction capability, the ammonia concentration of *ncd2* overexpression group decreased by 43.33% than that of without *Sphingomonas* sp. group, and decreased by 14.17% than that of *Sphingomonas* sp. Z392 group. In conclusion, *Sphingomonas* sp. Z392 might reduce the release of NH_3_ by reducing the flux of purine and arginine metabolisms, while enhancing ammonia assimilation to form nitrite. In this context, *ncd2* might be one of the key genes to reduce ammonia.

## 1 Introduction

The current environmental pollution in the livestock industry mainly comes from to feces, urine, sewage, noxious gases, noise, smoke, and dust (Ji et al., 2022), among them, the emission of malodor from livestock farms is also a major component of pollution in the animal husbandry industry. Studies have shown that large amounts of malodorous substances are generated during livestock farming and manure storage, posing a serious threat to both human and livestock health. These substances primarily include organic compounds, in addition to inorganic compounds, such as ammonia (NH_3_) and hydrogen sulfide (H_2_S), of which NH_3_ is particularly common and directly harmful. High ammonia concentrations in chicken coops jeopardize both human and animal health.

Currently, ammonia removal measures in chicken coops mainly include physical, chemical, feed regulation, and biological methods (Zhou and Wang, 2023). Physical methods include enhanced mechanical ventilation in the coop and the addition of physical deodorisers to the diet, which are often costly (Lubensky et al., 2019). Chemical methods include the use of inorganic metal ions, compounds, and acidifying agents, which tend to generate secondary pollution (Haq et al., 2021; Nancharaiah et al., 2016). The use of probiotics in poultry farming offers a promising approach to reducing ammonia emissions, improving animal health, and potentially promoting more sustainable and environmentally friendly agricultural practices (Chen and Ni, 2012). Microbial treatment techniques are not only characterized by strong selectivity, low cost, simple operation, high safety, non-toxic by-products, and lack of secondary pollution but can also lead to reductions in various environmental pollutants, thus positioning them as the optimal choice for bioremediation (Pang and Wang, 2021; Zhou and Wang, 2023). Different microorganisms have been reported to improve feed conversion efficiency, reduce fecal nitrogen levels in animals, and contribute to ammonia emission reduction, such as *Bacillus subtilis*, *Clostridium butyricum*, *Acinetobacter* (Huang et al., 2013), *Pseudomonas* (Jin et al., 2015), and *Agrobacterium* (Chen and Ni, 2012). However, few studies characterizing ammonia nitrogen-removing bacterial strains and their mechanisms have been conducted. In addition, previous studies have shown that adding *Sphingomonas* to broiler chicken feed significantly reduces ammonia concentrations in chicken coops during breeding (Wang et al., 2021). However, the mechanism of ammonia nitrogen reduction by *Sphingomonas* remains unclear. Therefore, to investigate the mechanism of ammonia removal in *Sphingomonas*, the transcriptome and metabolome of *Sphingomonas* were analyzed under different ammonium sulfate conditions, and the key genes in *Sphingomonas* associated with the reduction of ammonia release were screened. Subsequently, the effect of these key genes on the ability of *Sphingomonas* to reduce ammonia release was further investigated.

## 2 Materials and methods

### 2.1 Strains, media, and culture conditions

*Sphingomonas* sp. Z392 (GenBank accession no: MN108136) were isolated and stored at the Institute of Biology and Food Engineering of Huanghuai University. *Escherichia coli* β2155 was used for plasmid amplification.

*Sphingomonas* sp. Z392 and *E. coli* β2155 were cultured in Luriae–Bertani (LB) medium at 37°C. The heterotrophic nitrification medium (medium B) contained 0.5 g (NH_4_)_2_SO_4_, 5.62 g sodium succinate, and 50 mL Vicker’s salt solution (5 g K_2_HPO_4_, 2.5 g MgSO_4_⋅7H_2_O, 2.5 g NaCl, 0.05 g FeSO_4_⋅7H_2_O, 0.05 g MnSO_4_⋅4H_2_O, and 1 L distilled), distilled water 1.0 L. Moreover, media C and D were prepared by increasing the concentration of (NH_4_)_2_SO_4_ in medium B to 0.66 g/L and 1.06 g/L, respectively.

### 2.2 Transcriptome and metabolomics analysis

*Sphingomonas* sp. Z392 was cultured in LB medium at 37°C for 24 h. The cells were harvested by centrifugation (3,000 × *g*, 10 min) and washed with sterile saline, and the cell pellet was inoculated into medium B, incubated at 37°C. Then, the cells collected from medium B were added to medium C, incubated at 37°C. Finally, the cells from medium C were collected, transferred to medium D, incubated at 37°C. The cells from medium D were used for transcriptome and metabolome analysis.

Total RNA of the cells from medium D was extracted, transcriptome sequencing was performed by Gene *DeNovo* (Guangzhou, China) (Love et al., 2014). To obtain significant differentially expressed genes (DEGs), DESeq2 software was used with FC ≥ 2 or ≤ 0.5 (| log2(FC)| > 1) and *p* < 0.05 as screening conditions. Intracellular metabolites of the cells from medium D were detected by LC-MS, and metabolome were analyzed by Gene *DeNovo* (Guangzhou, China).

### 2.3 Strain construction

To screen antibiotics sensitive to *Sphingomonas* sp. Z392 as selection markers for genetic modification, the minimum inhibitory concentration (MIC) of gentamicin (Gm), streptomycin, and tetracycline against *Sphingomonas* sp. Z392 were determined. The experimental design for MIC using microplates were shown in Supplementary Tables 1, 2. For construction of *ncd2* gene overexpression plasmid, *ncd2* was amplified from the genomic DNA of Z392 strain. Then the PCR products were inserted into the expression plasmid p1600-gent-Flag with restriction sites *Nco*I and *Ecor*I through the Gibson assembly method, generating p1600-*ncd2*-Flag plasmid. The Z392 strain were transformed with 5 μg p1600-*ncd2*-Flag plasmid by electroporation (2.5kV/cm, 200 Ω, 5 ms).

To construct *ncd2* knockout strain, the targeting fragments (Δ*ncd2*:Gm) included the upstream and downstream homologous recombination arms of the *ncd2* gene, and were constructed by overlap PCR. Subsequently, The targeting fragment was cloned into the suicide plasmid pCVD442, resulting in the targeting plasmid pCVD442-Δ*ncd2*:Gm. The targeting vector pCVD442-Δ*ncd2*:Gm was then electrotransformed into the *E. coli* β2155 strain to generate the donor strain β2155/pCVD442-Δ*ncd2*:Gm. Finally, the *ncd2* gene knockout was achieved by conjugating the donor strain with the recipient strain (*Sphingomonas* sp. Z392), and the knockout of *ncd2* was verified by PCR.

### 2.4 Feeding and management of the experimental broiler chickens

A total of 1, 200 one- day-old Cobb broiler chickens with similar body weights (42 ± 2 g) were randomly divided into 4 groups: a control group (CK) without *Sphingomonas* sp. Z392, fed with *Sphingomonas* sp. Z392 group (WT), fed with Z392 OV*ncd2* group (OV), and fed with Z392 Δ*ncd2* (KO). The method refers to the research of Wang et al. (2021). At 7, 14, 21, 28, 35, and 42 days of age, fresh feces were collected from the broilers. The concentrations of total nitrogen, uric acid, ammonium nitrogen, and nitrate nitrogen in chicken manure were measured separately.

### 2.5 Nitrogen concentrations in chicken coops

The ammonia concentrations in the chicken coops were measured using an ammonia sensor (Bist et al., 2023). And total nitrogen, ammonia nitrogen, and nitrate nitrogen contents in chicken manure were measured (Such et al., 2023). Then, the determination of intracellular nitrite content was carried out using ion chromatography (Varão Moura et al., 2022).

### 2.6 Data and statistical analysis

All experiments were conducted in three biological replicates. The data were analyzed for statistical significance using SPSS 19.0 software. One-way analysis of variance (ANOVA) was used to express the significance of differences (*P* ≤ 0.05) between the groups. Figures were drawn using the GraphPad Prism 8 (GraphPad Software, USA).

## 3 Results

### 3.1 Transcriptomics analysis of *Sphingomonas* sp. Z392 under high level of ammonium sulfate

**FIGURE 1:**
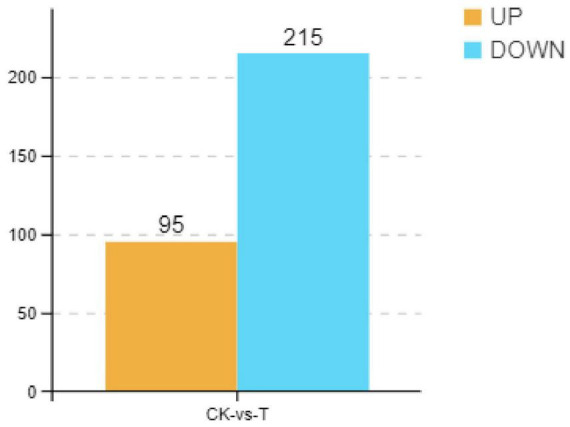
Differential expression genes of *Sphingomonas* sp. Z392 under high levels of ammonia sulfate. The *Y*-axis represents the number of up- and downregulated genes, orange represents upregulation, and blue represents downregulation.

Previous studies have demonstrated that *Sphingomonas* sp. Z392 can significantly reduce ammonia nitrogen level by converting ammonium nitrogen into nitrate nitrogen in chicken manure, decreasing the emission of ammonia from chicken feces. To explore the mechanisms of *Sphingomonas* sp. Z392 in reducing ammonia release, in this paper, transcriptomics analysis of *Sphingomonas* sp. Z392 cultured under high ammonium sulfate condition was performed. As shown in Figure 1, a total of 310 differentially expressed genes (DEGs) were identified between the high ammonium sulfate group and the control group (CK_vs_T), including 95 upregulated genes and 215 downregulated genes. Subsequently, the metabolic pathways enriched by the DEGs were further analyzed. As shown in Supplementary Figure 1, the DEGs were involved in the following pathways: ribosome, photosynthesis, drug metabolism, biosynthesis of some antibiotics, purine metabolism, oxidative phosphorylation, biosynthesis of secondary metabolites, monobactam biosynthesis, VB_6_ metabolism, pentose phosphate pathway, glutathione metabolism, cyanoamino acid metabolism, arginine biosynthesis, arginine and proline metabolism, amino acid biosynthesis, and nitrogen metabolism.

**FIGURE 2:**
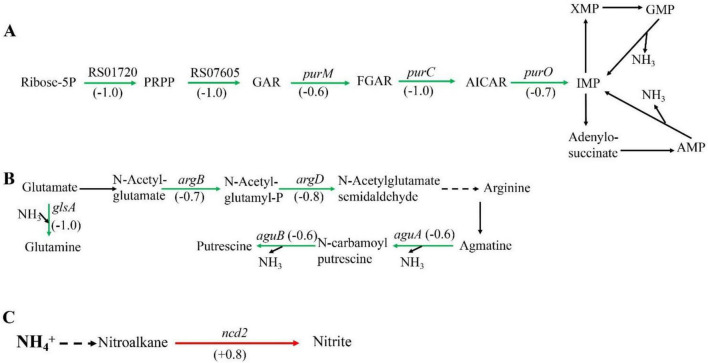
Significantly enriched metabolic pathways associated with ammonia degradation. **(A)** purine metabolism pathway. **(B)** Arginine metabolism pathway. **(C)** Nitrogen metabolism pathway. The green arrows indicate downregulation, and the red arrows indicate upregulation.

Among the metabolic pathways, amino acid metabolism, purine metabolism, and nitrogen metabolism are closely associated with nitrogen. Some amino acids can act as carriers of ammonia, such as arginine and glutamine. In addition, ammonia also exists in purines, which can release ammonia during purine metabolism. When *Sphingomonas* sp. Z392 was cultured under high concentration of ammonium sulfate, the purine metabolism, arginine biosynthesis, arginine and proline metabolism, and nitrogen metabolism pathways were significantly enriched. These pathways were closely associated with the reduce of ammonia (Figure 2).

In purine metabolism, ribose 5-phosphate accepts amino groups from glutamine, aspartate, and glycine to form inosine monophosphate (IMP), which serves as a carrier for the amino groups (Pedley and Benkovic, 2017). And, IMP can interconvert with adenylic acid (AMP) and guanosine monophosphate (GMP), accompanied by the release of NH_3_ (Gooding et al., 2015). As shown in Figure 2A, under high concentration of ammonium sulfate, the transcription levels of *RS01720*, *RS07605*, *purM, purC*, and *purO* genes in the purine metabolism of *Sphingomonas* sp. Z392 were significantly downregulated. As a result, the synthesis of IMP, GMP, and AMP might be reduced, which would lead to a decrease in the production of NH_3_ from the purine metabolism.

In addition, the biosynthesis and metabolism of arginine are also closely related to the intracellular ammonia concentration. Glutamate is converted to arginine via catalysis by a series of enzymes. Arginine, serving as a carrier of ammonia, undergo the arginine metabolism pathway to produce putrescine, and the ammonia is released (Landete et al., 2010). As shown in Figure 2B, under high concentration of ammonium sulfate, the transcription levels of *glsA*, *argB*, and *argD* genes of *Sphingomonas* sp. Z392 in arginine biosynthesis pathway were significantly downregulated, and the transcription levels of *aguB*, *aguA* genes in arginine metabolism pathway were also significantly downregulated.

In addition to purine and arginine metabolism, intracellular ammonia in *Sphingomonas* sp. Z392 might form nitromethane through a series of enzymes, then nitromethane is converted to nitrite via catalysis by a nitronate monooxygenase (encoded by the *ncd2* gene) (Figure 2C). When *Sphingomonas* sp. Z392 was cultured under high ammonium sulfate concentration, the transcription levels of *ncd2* in nitrogen metabolism pathway was significantly upregulated, which would facilitate the conversion of ammonia into nitrite, enhancing the elimination for intracellular ammonia.

### 3.2 Metabolomics analysis of *Sphingomonas* sp. Z392 under high level of ammonium sulfate

**FIGURE 3:**
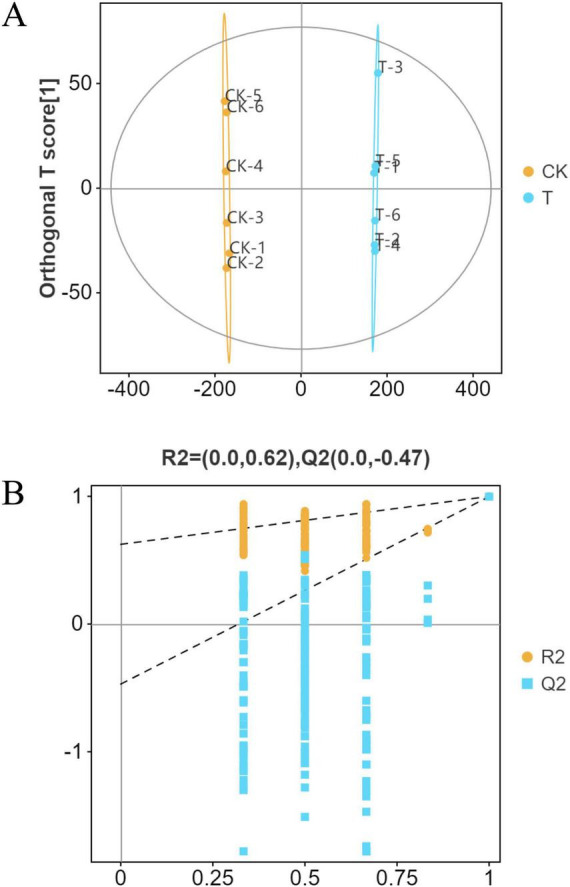
OPLS-DA score map **(A)** and displacement test **(B)** of metabolomics. OPLS-DA score plot and permutation test for the metabolome of *Sphingomonas* sp. Z392 under high levels of ammonia sulfate.

The metabolites of *Sphingomonas* sp. Z392 from the high ammonium sulfate and control groups were identified by LC-MS/MS. To analyze the changes in differential metabolites of *Sphingomonas* sp. Z392 under the high ammonium sulfate, the differential expression of metabolites (DEMs) between the CK and T groups were analyzed using orthogonal partial least squares-discriminant analysis (OPLS-DA). As shown in Figure 3, the clear separation of the profiles between the T and CK groups indicated that the model had a good predictive power, suggesting the existence of DEMs between the T and CK groups.

**FIGURE 4:**
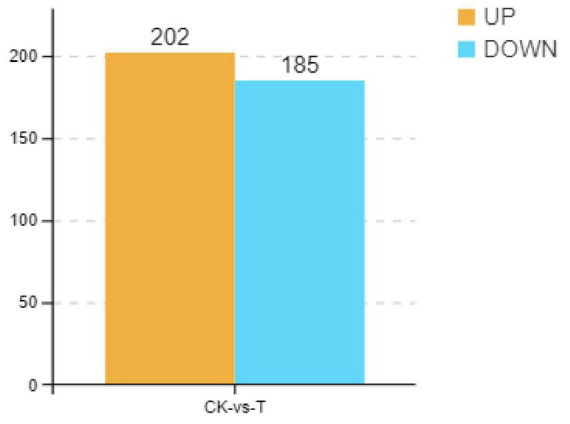
Differential expression metabolites of *Sphingomonas* sp. Z392 under high levels of ammonia sulfate., the *Y*-axis represents the number of up- and downregulated metabolites, orange represents upregulation, and blue represents downregulation.

**FIGURE 5:**
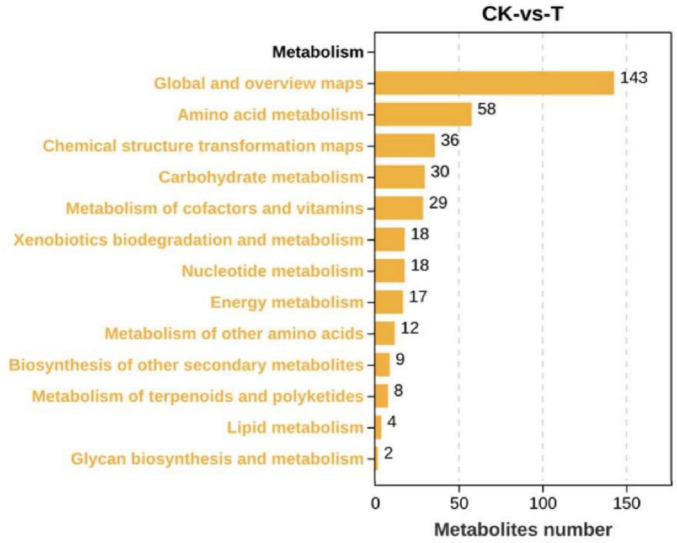
KEGG metabolic pathways enriched in differential expression metabolites.

As shown in Figure 4, there were 442 DEMs, of which 223 were significantly upregulated, and 219 were significantly downregulated when the *Sphingomonas* sp. Z392 was cultured under the high ammonium sulfate. Then, the KEGG metabolic pathways were enriched in DEMs. As shown in Figure 5, the DEMs were involved in the following pathways: amino acid metabolism, metabolism of other amino acids, nucleotide metabolism, carbohydrate metabolism, metabolism of cofactors and vitamins, biosynthesis of secondary metabolites, lipid metabolism, energy metabolism, xenobiotics biodegradation and metabolism, and metabolism of terpenoids and polyketides. Among these pathways, amino acid and nucleotide metabolisms are closely associated with nitrogen metabolism.

**FIGURE 6:**
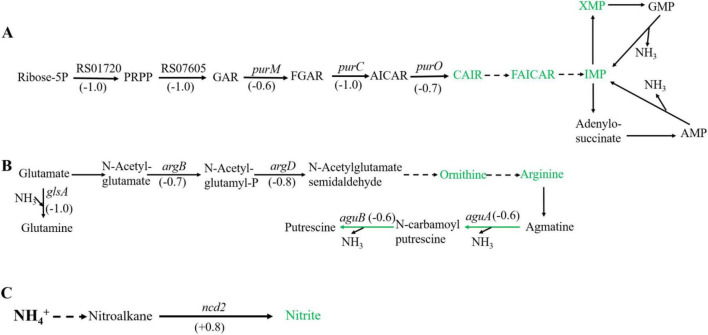
Differential expression metabolites in the purine metabolism **(A)** and arginine metabolism **(B)** pathways, and nitrogen metabolism pathway **(C)**. The green metabolites indicate downregulation, and the red metabolites indicate upregulation.

As shown in Figure 6, under high ammonium sulfate condition, the levels of carboxyaminoimidazole ribonucleotide (CAIR), 5-formamidoimidazole-4-carboxamide ribotide (FAICAR), IMP, and xanthosine monophosphate (XMP) of *Sphingomonas* sp. Z392 in purine metabolism were downregulated. In addition, under high ammonium sulfate condition, the levels of ornithine and arginine in the arginine metabolism pathway were downregulated. Therefore, under high concentration of ammonium sulfate, the concentration of arginine in *Sphingomonas* sp. Z392 might decrease, potentially leading to a reduced flux of arginine deamination to produce putrescine, which in turn decreases the production of NH_3_ from arginine decomposition.

**FIGURE 7:**
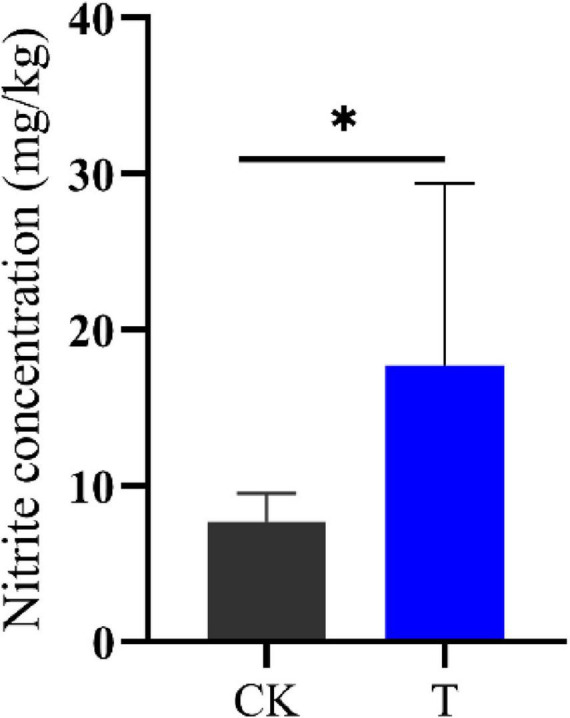
The nitrite concentrations of *Sphingomonas* sp. Z392 under conditions with (T) and without (CK) high levels of ammonia sulfate. * represents *p* < 0.05.

Intercellular nitrite and NH_3_ are closely related (Figure 2C), this study used ion chromatography to detect the intercellular nitrite content of *Sphingomonas* sp. Z392. Under high concentration of ammonium sulfate, the intercellular nitrite content of *Sphingomonas* sp. Z392 was 17.67 mg/kg, which was 2.27 times that of the *Sphingomonas* sp. Z392 without ammonium sulfate (7.77 mg/kg) (Figure 7).The above results showed that under high ammonium sulfate concentration, the transcription levels of *RS01720*, *RS07605*, *purM*, *purC*, and *purO* genes in the purine metabolism were significantly downregulated, and the corresponding levels of CAIR, FAICAR, IMP, and XMP of purine metabolism were also downregulated; In arginine biosynthesis and metabolism pathways, the transcription levels of *glsA*, *argB*, *argD*, *aguB*, and *aguA* genes were significantly downregulated, and the levels of ornithine and arginine were also downregulated. In addition, under high ammonium sulfate concentration, the transcription level of *ncd2* gene in nitrogen metabolism was significantly upregulated, and the intracellular nitrate content increased markedly.

### 3.3 Knockout and overexpression of the ncd2 gene in *Sphingomonas* sp. Z392

**FIGURE 8:**
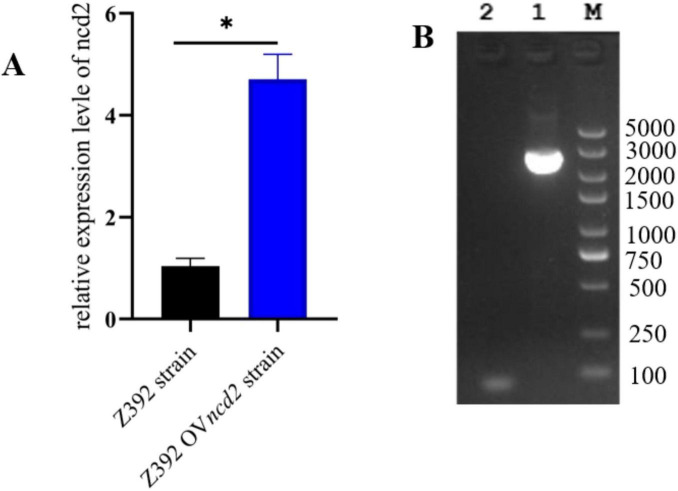
The verification of *ncd2* overexpression and knockout in *Sphingomonas* sp. Z392 by RT-qPCR **(A)** and PCR amplification **(B)**, respectively. Lane 1 represents band by the external primers, Lane 2 represents the negative band. M represents DL 5000 DNA Marker. * represents *p* < 0.05.

The above results demonstrated that the *ncd2* gene may contribute to ammonia reduction in *Sphingomonas* sp. Z392. To further explore the effect of the *ncd2* gene on the ammonia degradation ability of *Sphingomonas* sp. Z392, this study overexpressed and knocked out the *ncd2* gene in *Sphingomonas* sp. Z392, respectively, and named them Z392 OV*ncd2* and Z392 Δ*ncd2*. To validate the overexpression of *ncd2*, we performed quantitative RT-qPCR. As shown in Figure 8, the expression level of the *ncd2* in Z392 OV*ncd2* was significantly increased than that of the *Sphingomonas* sp. Z392. Furthermore, the genomic DNA of Z392 Δ*ncd2* was used as template, we amplified the target band with primers flanking *ncd2* and confirmed the successful knockout of the *ncd2* gene through sequencing.

### 3.4 Effect of *ncd2* on nitrogen content in chicken feces

**FIGURE 9:**
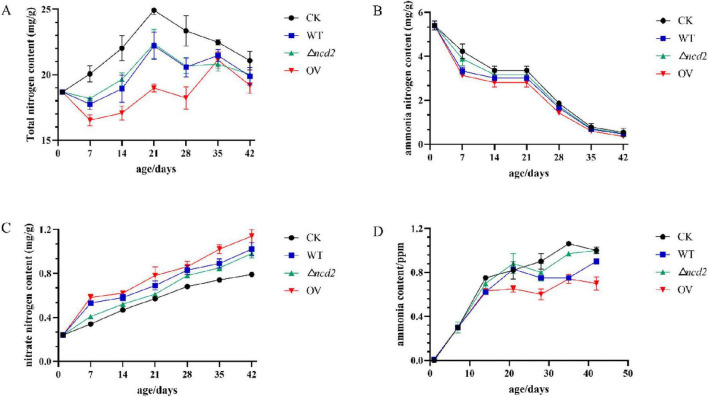
The changes of total nitrogen **(A)**, ammonium nitrogen **(B)**, nitrate nitrogen **(C)**, and ammonia **(D)** contents in chicken manure fed with *Spingomonas* sp. Z392, Z392 OV*ncd2*, Z392 Z392 Δ*ncd2*, and without *Spingomonas* sp.

To confirm the effect of *ncd2* on ammonia reduction, the broiler chickens were fed with *Sphingomonas* sp. Z392, Z392 OV*ncd2*, and Z392 Δ*ncd2* strains, respectively. Chicken manure was collected and tested for its contents of total nitrogen, ammonia nitrogen, and nitrate nitrogen (Figure 9).

When the broiler chickens were fed with *Sphingomonas* sp. Z392, Z392 OV*ncd2*, and Z392 Δ*ncd2* strains, the contents of total nitrogen and ammonium nitrogen in chicken manure were significantly decreased than that of without *Sphingomonas* sp., while nitrate nitrogen content was significantly increased than that of without *Sphingomonas* sp. Furthermore, Z392 OV*ncd2* strain exhibited the most ammonia reduction capability. The ammonia concentration of the group fed with Z392 OV*ncd2* decreased by 43.33% than that of without *Sphingomonas* sp. group, and decreased by 14.17% than that of group fed with *Sphingomonas* sp. Z392. In summary, overexpressing the *ncd2* gene in *Sphingomonas* sp. Z392 resulted in reduced ammonia emission and ammonium nitrogen content, while increasing nitrate nitrogen content in chicken coop. Overexpression of *ncd2* facilitated the conversion of ammonium nitrogen in chicken manure into nitrate nitrogen, thereby reducing ammonia emissions from chicken manure.

## 4 Discussion

Currently, biodegradation has received an increasing attention due to its advantages of low cost, high efficiency, simple operation, and environmental friendliness (Mi et al., 2019). The microorganisms reported to reduce ammonia mainly include *Alcaligenes faecalis*, *Pseudomonas*, *Rhodococcus*, *Acinetobacter*, and *Bacillus methylotrophicus* (Kim et al., 2005; Mi et al., 2019; Robertson and Kuenen, 1990; Zhao et al., 2010). However, the specific degradation mechanisms of these microorganisms toward ammonia are still unclear. *Sphingomonas* exhibits potential ammonia degradation ability due to its numerous pollutant degradation genes, but its systematic degradation pathway remains to be further studied.

In this study, *Sphingomonas* sp. Z392 was cultured under high ammonium sulfate environment, and transcriptome and metabolome analysis were performed jointly. It was found that the expression levels of genes related to purine metabolism and arginine metabolism in *Sphingomonas* sp. Z392 were decreased, and the levels of ornithine, arginine, IMP, and XMP were also downregulated. Purine metabolism and arginine metabolism are closely related to the intracellular ammonia concentration, under high ammonium sulfate environment, the flux of purine and arginine metabolisms might decrease, which might contribute to reduce NH_3_ release. Under high ammonium sulfate conditions, *Sphingomonas* sp. Z392 reduced the flux of purine metabolism, which not only helps to reduce ammonia release but also saves cellular energy, enhancing its survival ability in adverse environments. However, purine metabolism is closely related to the growth and reproduction of *Sphingomonas* sp. Z392, so the reduction of this metabolic flux can only be within a certain range, as excessive reduction may have adverse effects on cell growth and reproduction (Zhao et al., 2010). Arginine metabolism is an essential biochemical process for microorganisms to maintain normal growth and metabolism. This study found that under high ammonium sulfate conditions, the transcription levels of genes in arginine metabolism of *Sphingomonas* sp. Z392 decreased, resulting in reduced the levels of arginine and ornithine. Similarly, although the decrease in arginine metabolism flux could decrease NH_3_ release, this flux could only be reduced to a certain extent, otherwise it would affect the growth of *Sphingomonas* sp. Z392. Therefore, the reduction of flux in purine and arginine metabolism pathways might decrease ammonia release to some extent, but it cannot serve as the primary strategy. It is necessary to comprehensively consider the metabolic characteristics and growth requirements of *Sphingomonas* sp. to seek more effective and sustainable ammonia reduction solutions.

In addition, this study also found that under high ammonium sulfate environment, the transcriptional level of the *ncd2* in nitrogen metabolism pathway was upregulated, accompanied by a 2.27-fold increase in intracellular nitrite content. This pathway potentially converts NH_3_ into nitrite through a series of complex reactions, leading to a reduction in NH_3_ level. The *ncd2* encodes nitroalkane monooxygenase, which has been studied for the degradation of nitroalkanes (Gadda and Francis, 2010; Rolfes, 2006). After knocking out the *ncd2* gene, the ammonia concentrations in the chicken coop significantly increased, while the *ncd2*-overexpressing strain significantly reduced the ammonia concentration in the chicken coop, which was consistent with the previous studies (Vodovoz and Gadda, 2020; Nishino et al., 2010). Similar to the case of *Penicillium* and *Pseudomonas* aeruginosa, this enzyme is also involved in the metabolism of nitroalkanes, generating malondialdehyde, nitrite, and nitrate (Torres-Guzman et al., 2021). In this study, *Sphingomonas* sp. Z392 could increase the removal of ammonia by enhancing ammonia assimilation to form nitrite, which would become an effective pathway for ammonia degradation. Although, this study has discovered that *Sphingomonas* sp. Z392 might utilize the nitroalkane monooxygenase encoded by *ncd2* to convert nitromethane into nitrite, the specific pathway for ammonia to transform into nitromethane remains unknown. Current research suggested that completely ammonia-oxidizing bacteria have the potential for safe ammonia oxidation, which can directly oxidize ammonia to nitrate. The specific pathway for *Sphingomonas* sp. Z392 to convert ammonia into nitromethane still needs further exploration.

## 5 Conclusion

In this study, under high concentration of ammonium sulfate, the transcription levels of the genes related to purine metabolism and arginine metabolism pathways were significantly downregulated, and the corresponding levels of ornithine, arginine, IMP, and XMP were also downregulated in *Sphingomonas* sp. Z392. In addition, under high ammonium sulfate concentration, the transcription level of *ncd2* gene in nitrogen metabolism was significantly upregulated, and the intracellular nitrate content increased markedly. Under high concentration of ammonium sulfate, *Sphingomonas* sp. Z392 might reduce the release of NH_3_ by decreasing the flux of purine metabolism and arginine metabolism pathway, and increasing the assimilation flux of ammonia. Furthermore, *ncd2* was a key gene for ammonia reduction in *Sphingomonas* sp. Z392. In a word, this study not only systematically clarifies the metabolic pathway of ammonia reduction in *Sphingomonas*, but also points out future research directions.

## Data Availability

The datasets presented in this study can be found in online repositories. The names of the repository/repositories and accession number(s) can be found below: BioProject: Processed PRJNA1112986: the transcriptome of *Sphingomonas* sp. Z392 under high ammonium sulfate.
