# Supplementary material for: Multiomics-based analysis of the mechanism of ammonia reduction in Sphingomonas
Source: Front Microbiol. 2025 May 1;15:1437056. doi: 10.3389/fmicb.2024.1437056 (PMC12078293; doi:10.3389/fmicb.2024.1437056)
Supplement: Supplementary file 1 [file Data_Sheet_1.docx]

Supplementary Material

Multiomics-based analysis of the mechanism of ammonia reduction in *Sphingomonas*

Wang Mingcheng^1-4^, Liu Daoqi^4^, Xia Huili^4^, Wang Gailing^4^, Liu Chaoying^4^, Guo Yanan^5^, Guo Aizhen^1-3*^

^1^Country National Laboratory of Agricultural Microbiology

^2^ Country College of Veterinary Medicine

^3^ Hubei Hongshan Laboratory, Huazhong Agricultural University, Wuhan, Hubei, 430070, China

^4^College of Biological and Food Engineering, Huanghuai University, Zhumadian, Henan 463000, China

^5^Animal Science Institute, Ningxia Academy of Agriculture and Forestry Sciences, Yinchuan, Ningxia 750002, China

*** Correspondence:**

Guo Aizhen, College of Veterinary Medicine, Huazhong Agricultural University

E-mail: aizhen@mail.hzau.edu.cn

# Tables

Table S1 The design of MIC

| antibiotics | 1 | 2 | 3 | 4 | 5 | 6 | 7 | 8 | 9 | 10 | 11 | 12 |
| --- | --- | --- | --- | --- | --- | --- | --- | --- | --- | --- | --- | --- |
| Gentamicin | 200 | 100 | 50 | 25 | 12.5 | 6.25 | 3.13 | 1.56 | 0.78 | 0.39 | 0.2 | 0 |
|  | 200 | 100 | 50 | 25 | 12.5 | 6.25 | 3.13 | 1.56 | 0.78 | 0.39 | 0.2 | 0 |
| Streptomycin | 200 | 100 | 50 | 25 | 12.5 | 6.25 | 3.13 | 1.56 | 0.78 | 0.39 | 0.2 | 0 |
|  | 200 | 100 | 50 | 25 | 12.5 | 6.25 | 3.13 | 1.56 | 0.78 | 0.39 | 0.2 | 0 |
| Tetracycline | 200 | 100 | 50 | 25 | 12.5 | 6.25 | 3.13 | 1.56 | 0.78 | 0.39 | 0.2 | 0 |
|  | 200 | 100 | 50 | 25 | 12.5 | 6.25 | 3.13 | 1.56 | 0.78 | 0.39 | 0.2 | 0 |

Note: Each row represents different antibiotic concentrations, and the unit of antibiotic concentration is μg/mL.

Table S2 MIC experimental results

| concentrations（μg/mL） | 200 | 100 | 50 | 25 | 12.5 | 6.25 | 3.13 | 1.56 | 0.78 | 0.39 | 0.2 | 0 |
| --- | --- | --- | --- | --- | --- | --- | --- | --- | --- | --- | --- | --- |
| Gentamicin | + | + | + | + | + | + | - | - | - | - | - | - |
| Streptomycin | - | - | - | - | - | - | - | - | - | - | - | - |
| Tetracycline | + | + | + | + | + | + | + | + | + | - | - | - |

Note: "+" indicates inhibitory effect, "-" indicates no inhibitory effect.

## Supplementary Figures


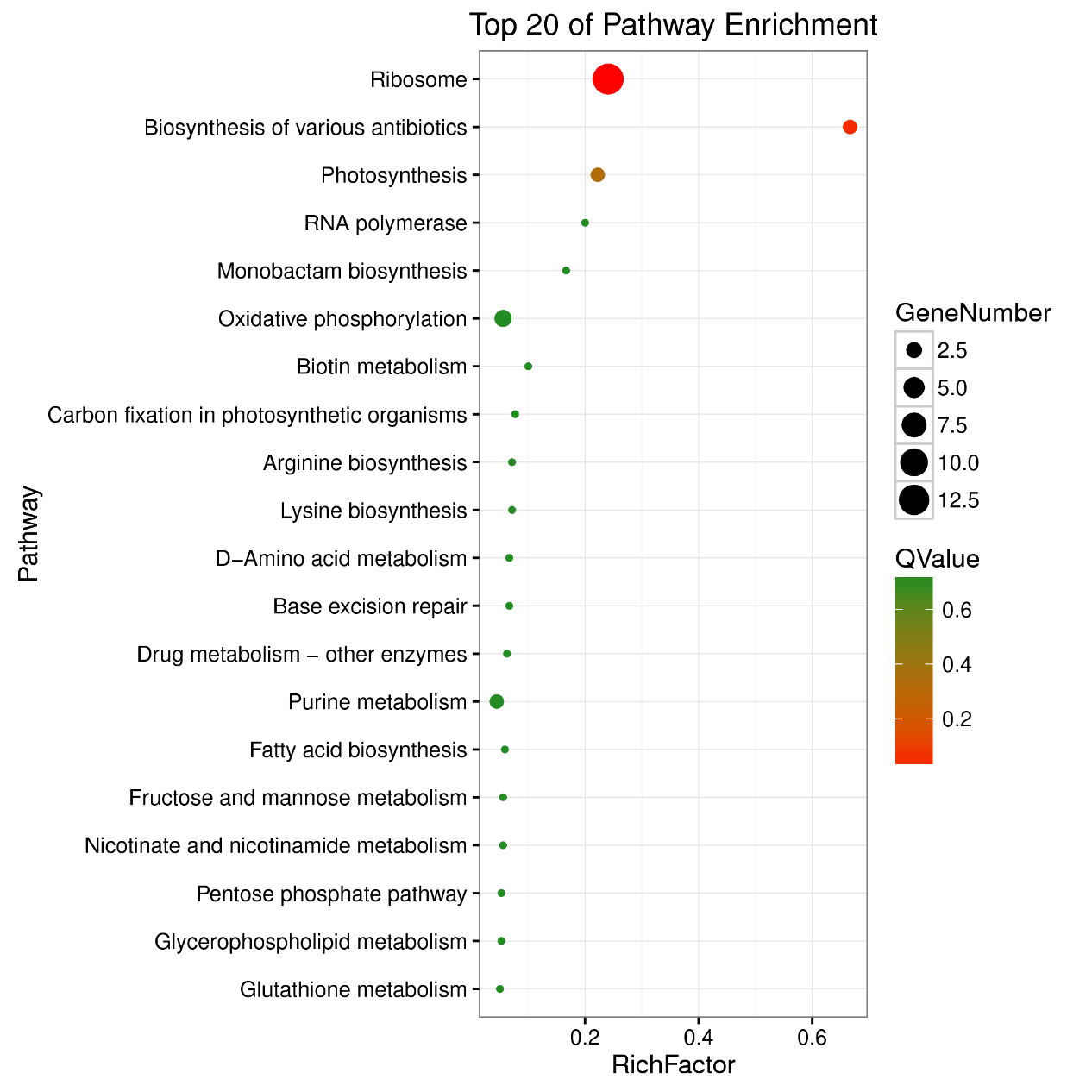


Figure 1S. The KEGG enrichment analysis of the DEGs
